# Protein expression, biochemical pharmacology of signal transduction, and relation to intraocular pressure modulation by bradykinin B_2_ receptors in ciliary muscle

**Published:** 2013-06-15

**Authors:** Najam A. Sharif, Shouxi Xu, Linya Li, Parvaneh Katoli, Curtis R. Kelly, Yu Wang, Shutong Cao, Rajkumar Patil, Shahid Husain, Laura Klekar, Daniel Scott

**Affiliations:** 1 Pharmaceutical Research, Alcon Research, Ltd., [a Novartis Company], Fort Worth, TX; 2 Medical University of South Carolina, Charlston, SC

## Abstract

**Purpose:**

To examine the bradykinin (BK) B_2_-receptor system in human and monkey ciliary muscle (CM) using immunohistochemical techniques, and to pharmacologically characterize the associated biochemical signal transduction systems in human CM (h-CM) cells. BK-induced modulation of intraocular pressure (IOP) in pigmented Dutch-Belt rabbits and cynomolgus monkeys was also studied.

**Methods:**

Previously published procedures were used throughout these studies.

**Results:**

The human and monkey ciliary bodies expressed high levels of B_2_-receptor protein immunoreactivity. Various kinins differentially stimulated [Ca^2+^]_i_ mobilization in primary h-CM cells (BK EC_50_=2.4±0.2 nM > Hyp^3^,β-(2-thienyl)-Ala^5^,Tyr(Me)^8^-(®)-Arg^9^)-BK (RMP-7) > Des-Arg^9^-BK EC_50_=4.2 µM [n=3–6]), and this was blocked by B_2_-selective antagonists, HOE-140 (IC_50_=1.4±0.1 nM) and WIN-63448 (IC_50_=174 nM). A phospholipase C inhibitor (U73122; 10–30 µM) and ethylene glycol tetraacetic acid (1–2 mM) abolished the BK-induced [Ca^2+^]_i_ mobilization. Total prostaglandin (primarily PGE_2_) secretion stimulated by BK and other kinins in h-CM cells was attenuated by the cyclooxygenase inhibitors bromfenac and flurbiprofen, and by the B_2_-antagonists. BK and RMP-7 (100 nM) induced a twofold increase in extracellular signal-regulated kinase-1/2 phosphorylation, and BK (0.1–1 µM; at 24 h) caused a 1.4–3.1-fold increase in promatrix metalloproteinases-1–3 release. Topical ocular BK (100 µg) failed to alter IOP in cynomolgus monkeys. However, intravitreal injection of 50 µg of BK, but not Des-Arg^9^-BK, lowered IOP in rabbit eyes (22.9±7.3% and 37.0±5.6% at 5 h and 8 h post-injection; n=7–10).

**Conclusions:**

These studies have provided evidence of a functional endogenously expressed B_2_-receptor system in the CM that appears to be involved in modulating IOP.

## Introduction

The endogenously produced nonapeptide (H-Arg-Pro-Pro-Gly-Phe-Ser-Pro-Phe-Arg-OH) bradykinin (BK) is generated by cleavage of the larger precursor polypeptide (kininogen) by specific proteases (kallikreins) within numerous tissues of the body [1]. The biologic actions of BK and Lys-BK are terminated when the kininase family of proteolytic enzymes degrades these peptides [1,2]. Two major BK receptor subtypes, namely, B_1_ and B_2_, mediate the functional effects of BK and Lys-BK [1-3]. Although the B_2_-subtype is found under normal physiologic conditions, the B_1_ subtype is typically induced during injury or trauma [1]. The B_1_ subtype has a low affinity for BK and a high affinity for Des-Arg^9^-BK; however, the B_2_ subtype exhibits a high affinity for BK and Lys-BK and a low affinity for Des-Arg^9^-BK [1]. Both receptor subtypes have been cloned from several species and have been shown to couple to G proteins and phospholipase C (PLC) to generate the second messengers inositol phosphates (IPs; including IP_3_) [4-7] and diacylglycerol (DAG) [3,7]. While IP_3_ mobilizes intracellular Ca^2+^ ([Ca^2+^]_i_), DAG phosphorylates protein kinase C, and together these events lead to the final biologic response such as tissue contraction, cell shape change, cell proliferation, fluid secretion, release of endogenous mediators, etc [3,7]. Additional events ensuing from elevation of [Ca^2+^]_i_ by BK include activation of nitric oxide synthase to produce nitric oxide (NO) that in turn activates guanylate cyclase to produce cyclic guanosine monophosphate (cGMP), and activation of cyclooxygenases (COX) to produce various endogenous prostaglandins (PGs) that in turn elevate intracellular cyclic adenosine monophosphate (cAMP) and/or activate the phosphoinositide (PI) hydrolysis cascade to further amplify the signal transduction pathways [3,7].

**Figure 1:**
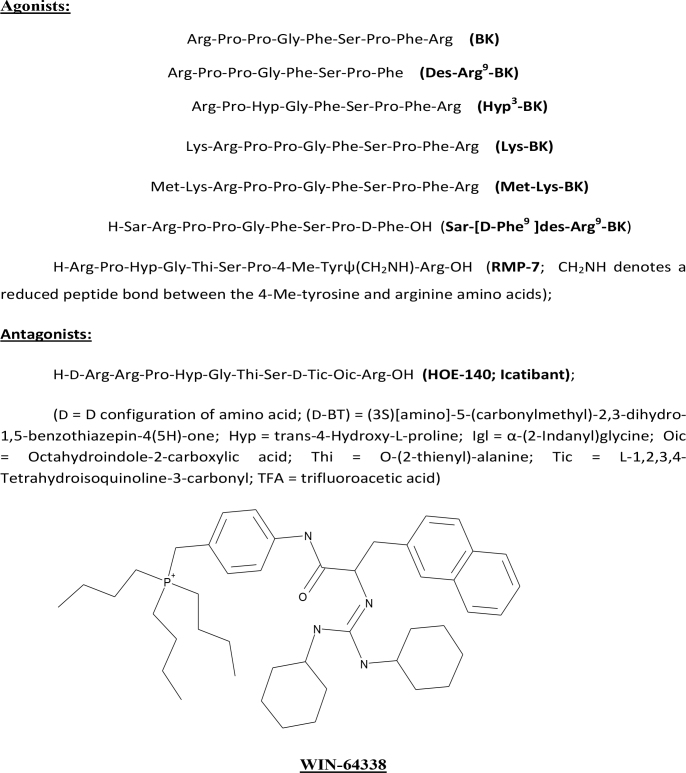
Chemical structures of the key compounds tested in the current studies. Peptide Agonists: Arg-Pro-Pro-Gly-Phe-Ser-Pro-Phe-Arg (BK); Arg-Pro-Pro-Gly-Phe-Ser-Pro-Phe (Des-Arg9-BK); Arg-Pro-Hyp-Gly-Phe-Ser-Pro-Phe-Arg (Hyp3-BK); Lys-Arg-Pro-Pro-Gly-Phe-Ser-Pro-Phe-Arg (Lys-BK); Met-Lys-Arg-Pro-Pro-Gly-Phe-Ser-Pro-Phe-Arg (Met-Lys-BK); H-Sar-Arg-Pro-Pro-Gly-Phe-Ser-Pro-D-Phe-OH (Sar-[D-Phe9 ]des-Arg9-BK); H-Arg-Pro-Hyp-Gly-Thi-Ser-Pro-4-Me-Tyrψ(CH2NH)-Arg-OH (RMP-7; CH2NH denotes a reduced peptide bond between the 4-Me-tyrosine and arginine amino acids). Peptide Antagonist: H-D-Arg-Arg-Pro-Hyp-Gly-Thi-Ser-D-Tic-Oic-Arg-OH (HOE-140; Icatibant). D = D configuration of amino acid; (D-BT) = (3S)[amino]-5-(carbonylmethyl)-2,3-dihydro-1,5-benzothiazepin-4(5H)-one; Hyp = trans-4-Hydroxy-L-proline; Igl = α-(2-Indanyl)glycine; Oic = Octahydroindole-2-carboxylic acid; Thi = O-(2-thienyl)-alanine; Tic = L-1,2,3,4-Tetrahydroisoquinoline-3-carbonyl; TFA = trifluoroacetic acid.

The ocular effects of BK have been studied to a relatively limited degree and include a confusing array of in vivo investigations in various animals related to the ability of BK to modulate intraocular pressure (IOP) [8-13] causing meiosis and inflammation [10,14]. Additional work has centered on perfusing bovine and human anterior segments with BK and monitoring outflow facility [15,16], detecting BK in aqueous humor [17] and in tears [18], and in vitro studies on the presence of mRNAs of the kallikrein/kinin system components (e.g., BK precursor; B_1_- and B_2_ receptor) [19], and signal transduction aspects in human trabecular meshwork (TM) and other cells [6,20-23]. However, little is known about the BK system in human ciliary muscle (h-CM). Therefore, the aims of the current studies were to characterize the kallikrein/kinin system in human CM tissues and cells, demonstrate the functional signal transduction pathways in h-CM cells, and characterize their pharmacology using various agonists and antagonists (Figure 1). We also compared certain aspects of the latter with human cloned B_2_ receptors expressed in Chinese hamster ovary (CHO-B_2_) cells.

## Methods

### Immunohistochemical determination of bradykinin receptors in ocular tissues

Since the human ciliary body contained a relatively high level of the mRNA for the B_2_ receptor [19], it was necessary to determine whether the cells in this tissue contained the respective B_2_-receptor protein. Thus, three human donor eyes obtained from a local eye bank and two cynomolgus monkey eyes (Covance Research Products; Madison, WI) were fixed in 4% alcoholic-zinc-paraformaldehyde fixative, processed into paraffin (CPI 09,013), and sectioned at 4 microns. Sections were antigen-retrieved and incubated with rabbit antihuman B_2_ receptor (Novus NLS797) or control rabbit immunoglobulin G (IgG; Jackson ImmunoResearch, West Grove, PA) overnight. Primary antibody labeling was detected with biotinylated donkey antirabbit IgG (Jackson ImmunoResearch) and streptavidin-horseradish peroxidase conjugate (DAKO, Carpinteria, CA). Labeling was developed with 3-amino-9-ethylcarbazole (AEC), a high sensitivity peroxidase substrate (DAKO). Sections were counter-stained with hematoxylin. Unfortunately, suitable antibodies for the rabbit B_2_ receptor are not commercially available; thus, similar studies could not be conducted on rabbit eye sections to correlate with the IOP studies described in the following sections.

### [^3^H]-BK binding to human cloned B_2_ receptor

To first define the B_2_-receptor binding affinity of the key BK-related peptides for subsequent concentration selection for cell-based experiments, we first decided to conduct radioligand binding experiments. However, in view of the difficulty of obtaining sufficient h-CM cells for these initial studies, we decided to use cell membranes of Chinese hamster ovary cells containing the human cloned B_2_ receptor (CHO-B_2_; Euroscreen, Gosselies, Belgium). Thus, CHO-B_2_ cell homogenates (2.5 µg protein) were incubated for 60 min at 23 °C with 0.2 nM [^3^H]-BK (95.5 Ci/mmole; PerkinElmer, Cambridge, MA) in the absence or presence of the test compound in a buffer containing 50 mM Tris/HCl (pH 7.4), 0.2 g/l 1–10-phenanthroline, and 0.1% bovine serum albumin [5,21]. Non-specific binding was determined in the presence of 1 µM BK. Following this incubation, the samples were filtered rapidly under vacuum through glass fiber filters (PerkinElmer) presoaked with 0.3% polyethyleneimine and rinsed several times with an ice-cold 50 mM Tris-HCl buffer using a 96-sample cell harvester (TomTec; Gaithersburg, MD). The filters were air-dried and the radioactivity counted in a beta-scintillation counter (TopCount, PerkinElmer). Data were analyzed using a sigmoidal-fit, iterative algorithm of a computer program designed to automatically fit the new data (Activity base; IDBS, Surrey, UK) [5,21]. The equilibrium inhibition constants (K_i_, drug concentration required to inhibit the binding by 50%) were calculated and the mean±standard error of the mean (SEM) values determined thereafter.

### Functional assay measuring [Ca^2+^]_i_ mobilization in cultured human ciliary muscle cells

Since the mRNA and protein immunoreactivity for the B_2_ receptor was confirmed to be present in human h-CM, it was deemed important to demonstrate that functionally coupled and pharmacologically sensitive B_2_ receptors existed in this tissue. Accordingly, we isolated h-CM cells and studied BK-induced [Ca^2+^]_i_ mobilization in these cells using the Fluorescence Imaging Plate Reader (FLIPR; Molecular Devices, Menlo Park, CA) instrument as previously described [24-27]. Low passage h-CM cells were seeded at a density of about 20,000 cells/well in a black-wall, 96-well tissue culture plates and grown for 2 days. On the day of the experiment, one vial of FLIPR Calcium Assay Kit dye (Molecular Devices) was resuspended in 50 ml of a FLIPR buffer consisting of Hank’s Balanced Salt Solution (HBSS), 20 mM HEPES, and 2.5 mM probenecid, pH 7.4. Cells were loaded with the calcium-sensitive dye (proprietary to Molecular Devices) by adding an equal volume (50 µl) to each well of the 96-well plate and incubated with dye for 1 h at 23 °C. Then the test compound plate and the cell plate were placed in the FLIPR instrument, and an aliquot (25 μl) of the BK solution (positive control) or the test compound was added to the existing 100 μl dye-loaded cells at a dispensing speed of 50 μl/sec. Fluorescence data were collected in real time over a period of time. Responses were measured as peak fluorescence intensity minus basal and where appropriate are expressed as a percentage of a maximum BK-induced response [E_max_ %]. When antagonist studies were performed, the latter were incubated with the cells for 15 min before BK was added. When the effects of various cellular signaling inhibitors/treatments were investigated, they were added to the cells 5 min before BK was added. The potencies (EC_50_ or IC_50_ values) of agonists and antagonists were determined from concentration-response curves using a sigmoidal-fit, iterative algorithm of a computer program designed to automatically fit the raw data (ActivityBase, IDBS, Surrey, UK). The relative intrinsic activities (E_max_ values) of the agonists were determined relative to reference agents where the maximal response was set to 100%. Concentration-response curves and other plots for the manuscript were constructed using Origin® software package (Microcal® Software Inc., Northampton, MA) [24-27].

### Measurement of prostaglandins in human ciliary muscle and Chinese hamster ovary cells expressing B_2_ receptors

Since functionally coupled B_2_ receptors were demonstrated in h-CM cells, we decided to study a downstream event from the [Ca^2+^]_i_ mobilization that BK and related peptides may induce. This involved stimulating h-CM cells and CHO-B_2_ cells with various concentrations of test peptides and measuring the release of various PGs in the extracellular medium. The concentration-dependent effects of these peptides on total PGs, and specifically PGE_2_, PGF_2α_, and PGD_2_ release, were studied. Cells were grown as a monolayer in a 24-well plate (precoated with fibronectin) to confluence and subsequently serum-starved overnight before the experiment. On the day of the experiment, just before the drug was added, the wells were aspirated completely, and 990 µl basal medium and 10 µl 100× series of concentrations of the different peptide agonists were added to achieve the desired final concentrations of each compound (e.g., 0.1 nM to 10 µM). Plates were incubated with BK agonists for 1 h at 37 °C in the incubator. At the end of the incubation period, the cellular supernatants were collected and stored at −80 °C or used immediately to determine the PG content. Cell supernatants were tested for total PG levels and sub-species of PGs (PGE_2_, PGF_2α_, and PGD_2_) using specific competitive enzyme immunoassays (Cayman Chemical Co., Ann Arbor, MI) as directed in the manufacturer’s recommended protocols using the supplied standards and reagents. The limits of detection for various PGs were as follows: total PGs=29 pg/ml; PGE_2_=15 pg/ml; PGF_2α_=9 pg/ml; PGD_2_=200 pg/ml.

### Measurement of nitric oxide production

In certain cell types, BK elevates intracellular levels of nitric oxide (NO), which in turn causes the generation of cGMP via the soluble guanylate cyclase in the cell cytosol [3,28]. In the present studies, we determined whether the B_2_ receptors in h-CM cells were coupled to NO production using a specific kit (Promega Corp., Madison, WI) and according to the manufacturer’s instructions.

### Measurement of extracellular signal-regulated kinase-1/2 phosphorylation and pro-matrix metalloproteinase production

Since BK has been shown to stimulate ERK1/2 phosphorylation in h-TM cells as an early step in the release of matrix metalloproteinases (MMPs) [23], we sought to ascertain whether a similar phenomenon was operable in h-CM cells. In the current assay, an homogeneous-time-resolved fluorescence (HTRF) assay (CisBio; Bedford, MA) was used. The HTRF assay is based on a sandwich immunoassay using an antiphospho-ERK1/2 antibody labeled with D_2_ and an anti-ERK1/2 antibody labeled with Eu^3+^-Cryptate. In brief, h-CM cells (passage 7) were cultured in 96-well half-area white plates and incubated overnight, at 37 °C in CO_2_ atmosphere. Cells were then incubated in a serum-free culture medium overnight before being exposed to BK or vehicle at 23 °C. Lysis buffer provided in the HTRF kit was used to lyse the cells at 23 °C with shaking for 30 min after the agonist treatment. HTRF conjugates containing phospho-ERK1/2 antibodies were then added to the lysates and incubated for an additional 2 h at 23 °C. The fluorescence signal was recorded using a Tecan M1000 fluorescence plate reader (Molecular Devices) at 620 nm for the donor and 665 nm for the acceptor.

The release of pro-MMP-1/2/3 in the extracellular medium of h-CM cells exposed to kinin’s, for 24 h at 37 °C [29,30] was studied next. Thus, h-CM cells isolated from multiple human donor eyes were cultured as described above and then serum-starved for 24 h prior to the addition of any test compound. Cells were incubated with the vehicle or test compound for 24 hours at 37 ºC after which the medium was collected and concentrated ten-fold using Centricon concentrators (Millipore Corp., Bellireca, MA). Equal volumes of this concentrate were loaded on 10% SDS-polyacrylamide gels (SDS-PAGE) and the proteins separated and transferred to nitrocellulose membranes. The membranes were then blocked with 5% non-fat dry milk (Bio-Rad Laboratories, Hercules, CA) followed by incubation with antibodies to pro-MMP-1, pro-MMP-2, or pro-MMP-3 for 12 hours at 4ºC with gentle shaking. After washing, these membranes were incubated with appropriate secondary antibodies (HRP-conjugated; dilution 1:3000) for 1 hour at 20 ºC. The pre-stained proteins and molecular weight markers were run in parallel. For chemiluminescent detection, the membranes were treated with enhanced chemiluminescent reagent (Amersham Pharmacia, Piscataway, NJ) and the signal was quantified using a Bio-Rad Versadoc imaging system (Bio-Rad Laboratories, Hercules, CA) [29,30].

### Intraocular pressure measurements

To extend our in vitro observations of the high density of B_2_-receptor protein in human and cynomolgus monkey ciliary muscle and non-pigmented ciliary epithelium, we studied the effects of topically administered BK (100 µg total dose in a sterile vehicle) on IOP in normotensive and TM-ablated hypertensive eyes of these animals [30,31]. Since no remarkable changes in IOP were observed, we concluded that most likely the peptide did not penetrate the cornea to reach the anterior chamber. However, since we could not subject the monkeys to additional studies involving BK delivery to the eye interior, it was decided that intravitreal (ivt) injection of BK into pigmented Dutch-Belt rabbits would be worth pursuing. Thus, after light corneal anesthesia with 0.1% proparacaine, basal IOPs were determined in up to 10 Dutch-Belt rabbits using an Alcon Pneumotonometer (Alcon Laboratories Inc., Fort Worth, TX) [30,31]. After this time, 50 µg of BK (B_2_ agonist) or Des-Arg^9^-BK (B_1_ agonist) in 20 µl of sterile basal salt solution (BSS plus), or just BSS plus vehicle, was injected ivt into one eye of each rabbit and the IOP determined at various times thereafter. Authors confirm adherence to the ARVO Statement for Animals in Ophthalmic and Vision Research, with all animal studies conducted according the guidelines for Animal Care and Use at Alcon Research Ltd.

## Results

### B_2_-receptor immunohistochemistry

**Figure 2:**
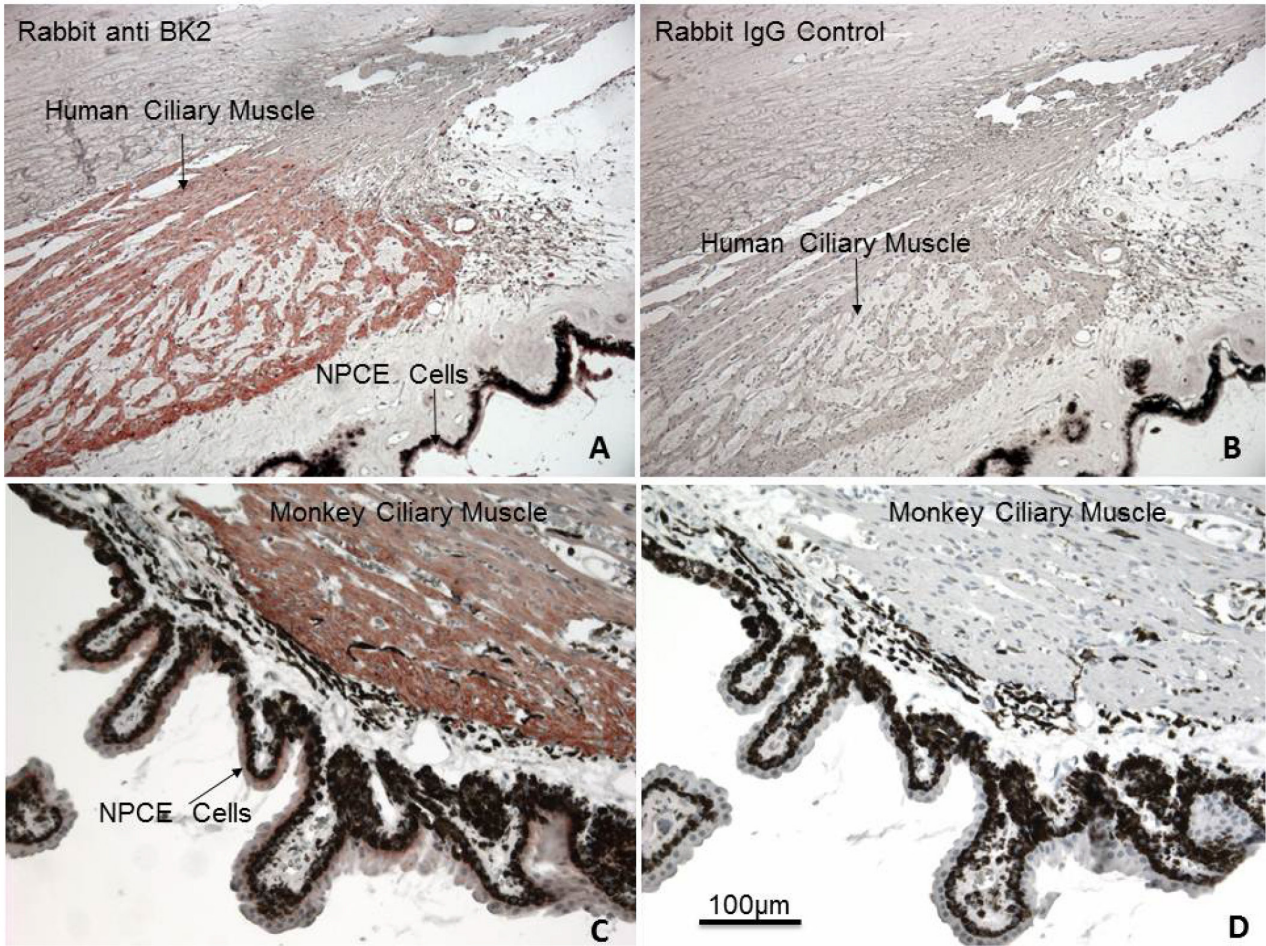
Localization of B_2_-receptor immunoreactivity in ocular tissues of human and cynomolgus monkey eyes. Section **A** shows robust labeling of human ciliary muscle (h-CM) fibers and NPCE cells, while section **B** is the negative control (IgG control) for human eye sections. The labeling of monkey CM fibers and NPCE cell B_2_ receptors is evident in section **C**. Once again, no specific labeling was observed in the IgG control section of the monkey eye (section **D**). The scale bar shown in **D** (100 μm) applies to all the figures.

The protein encoded by the mRNA for the B_2_-receptor subtype was examined with immunohistochemical procedures conducted on thin sections of postmortem human and cynomolgus monkey eyes. A high level of B_2_-receptor protein immunoreactivity was expressed by human (Figure 2A,B) and monkey ciliary bodies (ciliary muscle and non-pigmented ciliary epithelium; Figure 2C,D). The negative control experiments conducted on adjacent sections of the same eyes using IgG control showed no specific labeling of the B_2_-receptor protein (i.e. sections B and D for each species).

### Mobilization of [Ca^2+^]_i_

**Figure 3:**
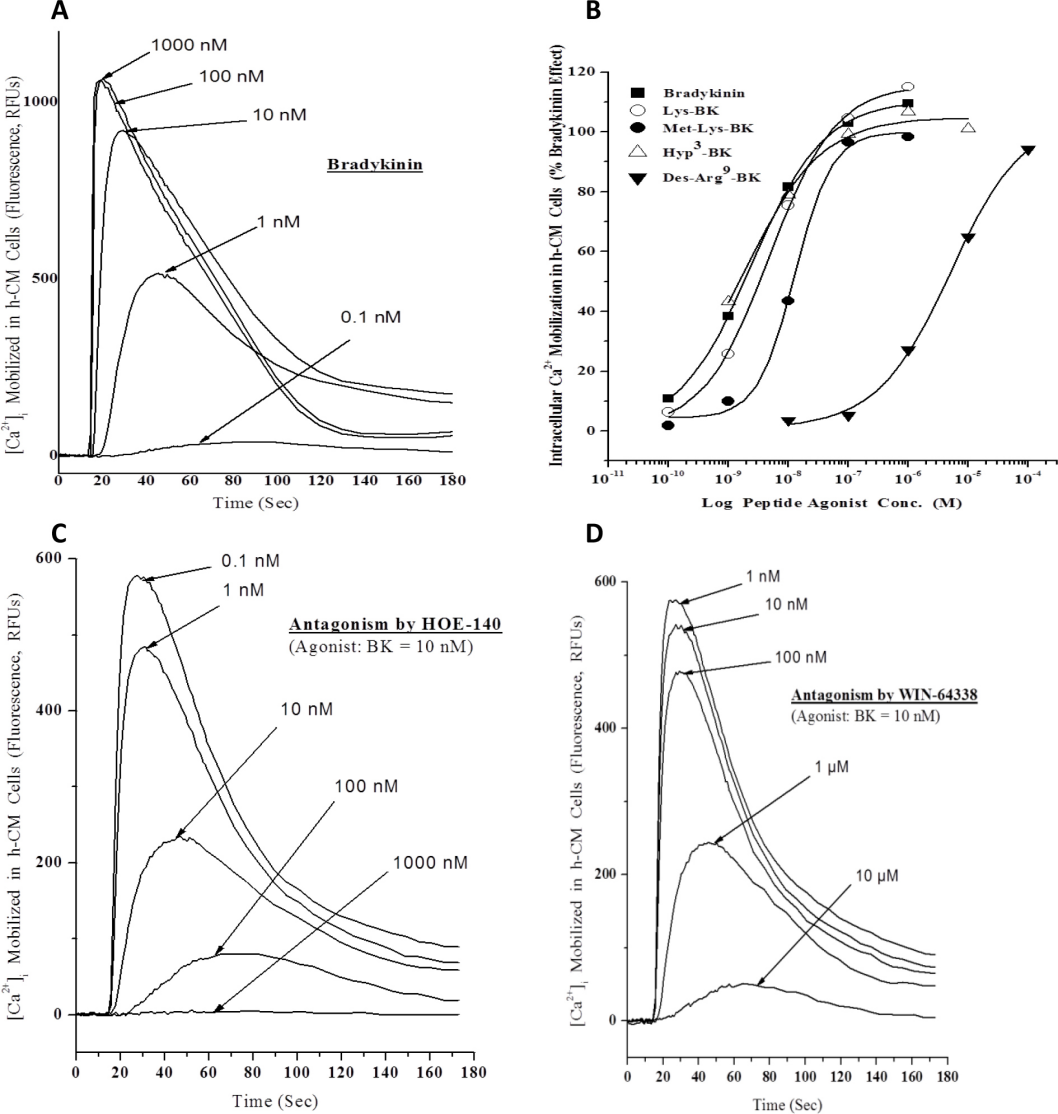
Concentration-dependent effect of bradykinin (BK) on mobilization of [Ca^2+^]_i_ in normal, primary h-CM cells. Fluorescent dye-loaded h-CM cells were exposed to different concentrations of BK and the changes in relative fluorescence units (RFUs), indicating changes in levels of [Ca^2+^]_i_, monitored over time (Figure 3A). The peak responses to various BK concentrations were then used to construct the concentration-response curves. Other BK-related peptides were tested in the same manner and their concentration-response curves plotted (e.g., data from a representative experiment; Figure 3B). Agonist potency data from many such experiments were obtained and are shown in Table 1 as mean ± SEM of EC_50_ values (n=3–7). The effects of a peptide B_2_-receptor antagonist (HOE-140; Figure 3C) and a non-peptide antagonist (WIN-64338; Figure 3D) on BK (10 nM)-induced elevation of [Ca^2+^]_i_ were also determined, and antagonist potencies derived (Table 1, lower section).

To determine whether the B_2_-receptor subtype immunoreactivity observed in human CM tissue (Figure 2A) related to functionally active receptors, we isolated h-CM cells from multiple human donor eyes and performed cell-based functional assays to monitor increases in [Ca^2+^]_i_ in response to BK and the related family of peptides. Accordingly, h-CM cells exhibited elevated levels of mobilized [Ca^2+^]_i_ when challenged with different concentrations of these peptides, for example, BK itself (Figure 3A). The collated concentration-response data obtained from many such studies (e.g., Figure 3B) indicated the following rank order to potency of these compounds: Hyp^3^-BK EC_50_=2.2±0.2 nM=BK EC_50_=2.4±0.2 nM > Lys-BK EC_50_=3.2±0.8 nM=RMP-7 EC_50_=3.7±1.2 nM > Met-Lys-BK EC_50_=16.1 nM >> Des-Arg^9^-BK EC_50_=4.2 µM (Table 1). The two BK receptor antagonists, HOE-140 (IC_50_=1.4±0.1 nM; Figure 3C) and WIN-64338 (IC_50_=174±18 nM; Figure 3D), abrogated the BK-induced responses in h-CM cells with potencies (Table 1, second column) that matched their affinities for the B_2_ receptor determined by [^3^H]-BK binding to the cloned human B_2_ receptor expressed in the cell membranes of CHO-B_2_ cells (Table 1, last column).

**Table 1:** Functional agonist and antagonist potencies at primary h-CM cell B_2_-receptor compared with h-TM cell responses, and ligand binding to human cloned B_2_-receptors

**Compound**	**Agonist Potency at stimulating [Ca^2+^]_i_ mobilization in primary h-CM cells** **(EC_50_, nM)**	***Agonist Potency at stimulating [Ca^2+^]_i_ mobilization in primary h-TM cells*** ***(EC_50_, nM)***	**Ligand binding inhibition constant at human cloned B_2_ receptors** **(K_i_, nM)**
**Agonists**			
Hyp^3^-BK	2.2±0.2	*1*	1.9±1.1
BK	2.4±0.2	*1*	0.5±0.01
Lys-BK	3.2±0.8	*2*	1.8±0.7
RMP-7	3.7±1.2	*nd*	11.3±1.1
Met-Lys-BK	16.1±6.1	*7*	73.0±24.7
Des-Arg^9^-BK	4,200±570	*3,600*	10,400±5,900
**Antagonists blocking the actions of BK or competing for [^3^H]-BK binding to B_2_-receptor**	**Antagonist potency (IC_50_, nM)**	***Antagonist potency*** ***(IC_50_, nM)***	**Ligand binding inhibition constant at human cloned B_2_ receptors** **(K_i_, nM)**
HOE-140	1.4±0.1	*5*	0.5±0.2
(S)-WIN-64338	174±18	*270*	170.0±52.2

The [Ca^2+^]_i_ mobilization data in h-CM cells are mean ± SEM from 3 to 7 experiments. The [^3^H]-BK binding data are from up to 19 experiments. Preliminary h-TM cell data are shown for comparison purposes where the potency values have been rounded-off for clarity. The pharmacology of the h-CM and h-TM cell [Ca^2+^]_i_ mobilization data was well correlated: r=0.99, p<0.0001, n=7. Also, the human cloned B_2_-receptor binding pharmacology and h-CM [Ca^2+^]_i_ mobilization data correlated well: r=0.99, p<0.0001, n=8; nd represents not determined.

**Figure 4:**
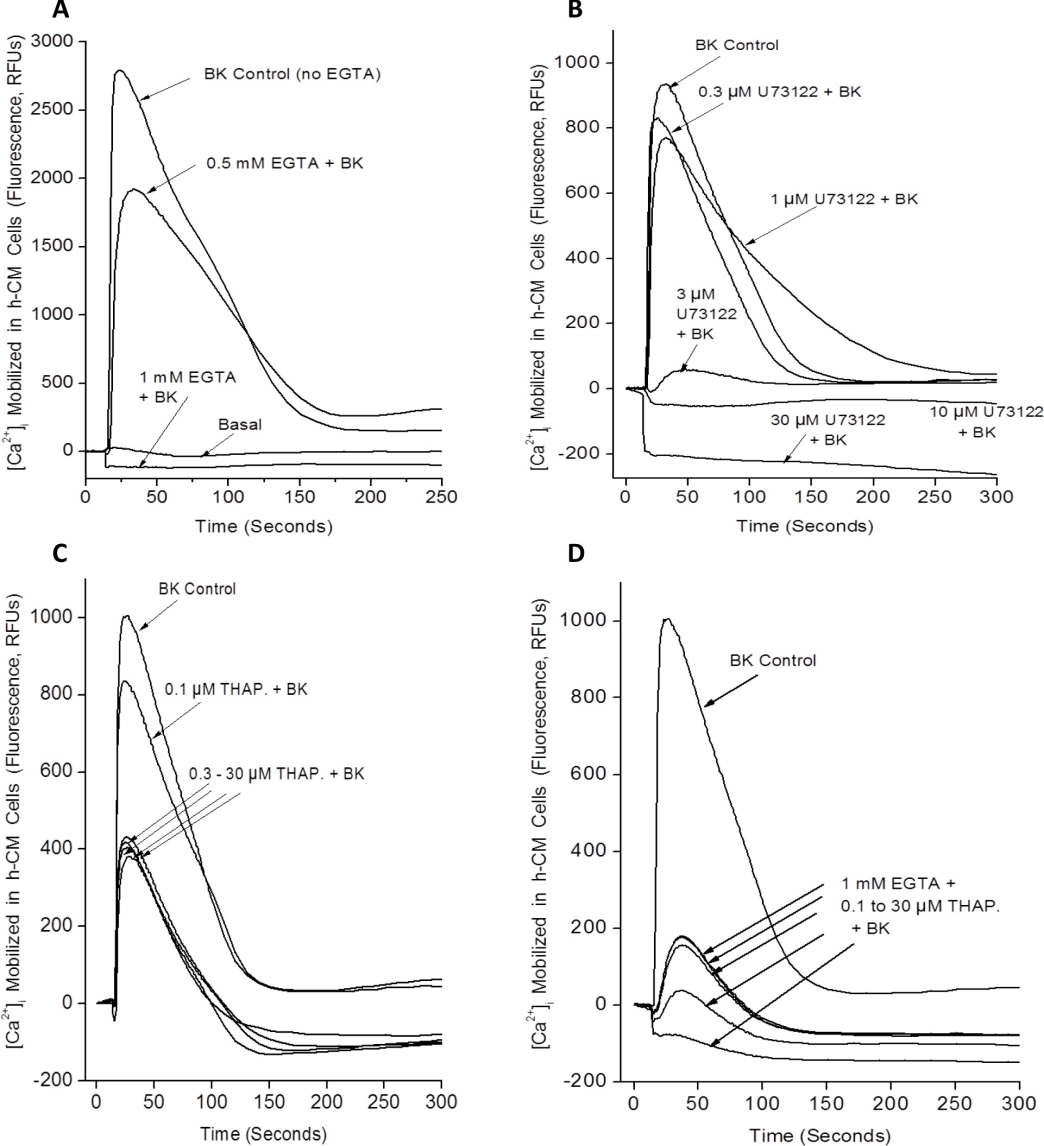
Effects of various inhibitors of cellular signaling on bradykinin (BK)-induced mobilization of [Ca^2+^]_i_ in normal, primary h-CM cells. The effect of removing extracellular Ca^2+^ (by chelation with different concentrations of EGTA) relative to the BK-induced response in the absence of EGTA is shown in **A**. The effect of preincubating cells with different concentrations of the PLC inhibitor U73122 on BK-induced [Ca^2+^]_i_ levels compared to control cells is shown in **B**. Figure **C** depicts [Ca^2+^]_i_ mobilization evoked by BK in control and thapsigargin pretreated h-CM cells. Figure **D** shows BK-induced mobilization of [Ca^2+^]_i_ in h-CM cells preincubated with 1 mM EGTA and with different concentrations of thapsigargin and that observed in control cells.

In subsequent experiments, we used various inhibitors of cell signaling and other treatments to determine the source of the [Ca^2+^]_i_ in h-CM cells detected in the FLIPR Tetra experiments. Preincubating h-CM cells with ethylene glycol tetraacetic acid (EGTA) for 5 min to chelate extracellular Ca^2+^ before BK was added caused a diminution of the [Ca^2+^]_i_ response, with complete abolition of the response to BK with 1 mM EGTA (Figure 4A). Exposing h-CM cells to a PLC inhibitor (U73122; 0.1 nM to 10 µM) before BK (100 nM) was added resulted in a concentration-dependent reduction in the Ca^2+^-mobilizing effects of BK (Figure 4B), indicating that the Ca^2+^ monitored was originating, at least partially, from the endoplasmic reticulum (ER) and was being mobilized by the IP_3_-generation pathway [7]. Indeed, when the ER pool of Ca^2+^ was depleted by preincubating h-CM cells with thapsigargin (0.1 or 30 µM), the [Ca^2+^]_i_ levels mobilized by BK were reduced by >50% compared to the control cells that had not been exposed to thapsigargin (Figure 4C), thus suggesting an alternative source of Ca^2+^ in addition to the ER possibly involving entry via Ca^2+^ channels on the cell membrane. Interestingly, when extracellular Ca^2+^ was depleted when 1 mM EGTA was included, BK continued to raise [Ca^2+^]_i_ by a small amount, and this was also concentration-dependently reduced by thapsigargin (Figure 4D). Since the ER also contains a ryanodine-sensitive Ca^2+^ channel, in addition to the IP_3_-sensitive one [32], we were interested to determine whether adding ryanodine to h-CM cells enhanced the mobilization of [Ca^2+^]_i_ above and beyond that could be induced by BK itself. However, ryanodine (1–30 µM) did not enhance but instead slightly reduced the effect of BK on the [Ca^2+^]_i_ levels (data not shown).

### Production and release of prostaglandins

**Figure 5:**
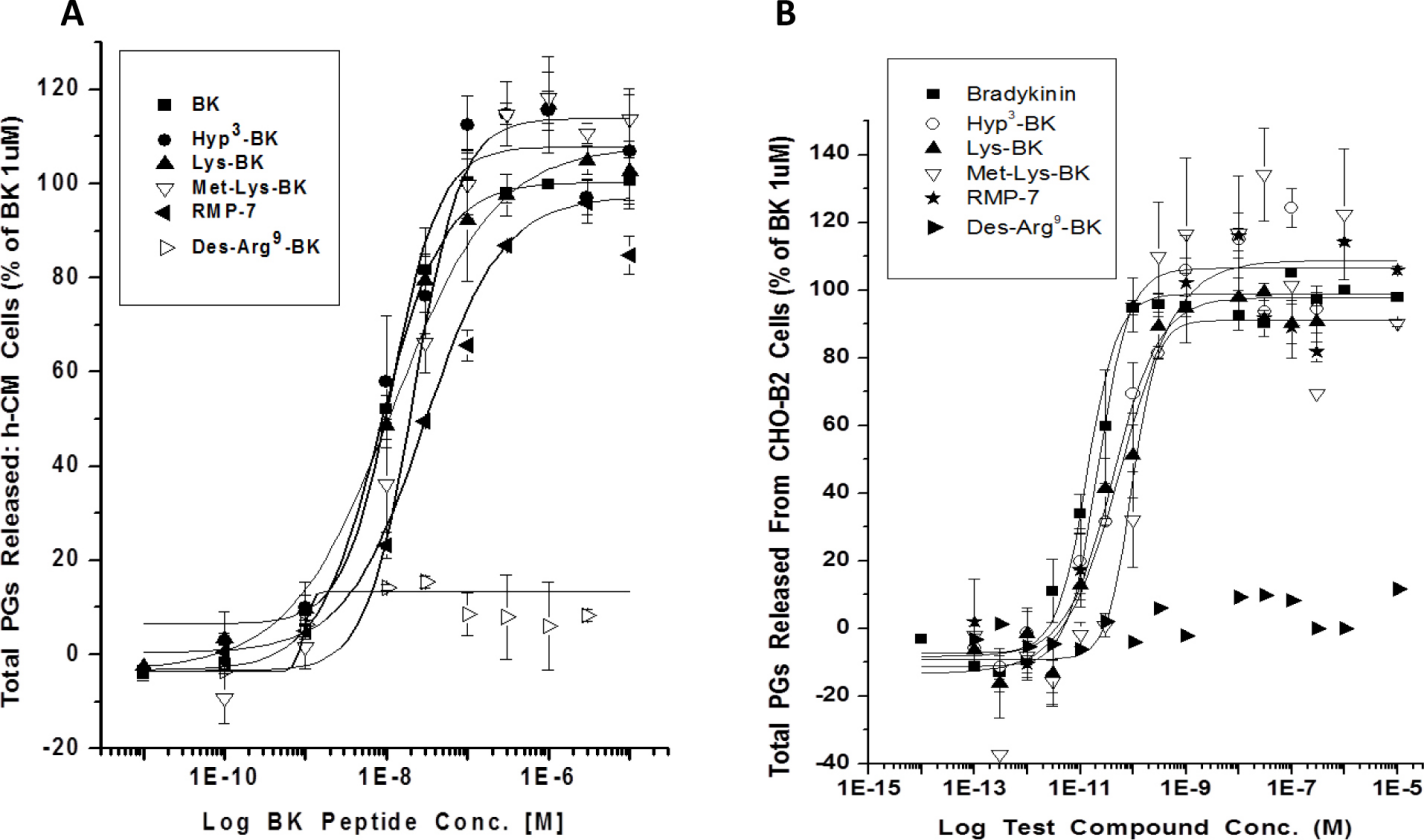
Effect of bradykinin (BK) and related peptides on total PG synthesis and secretion in h-CM and CHO-B_2_ cells. **A**: Concentration-dependent effects of various kinin peptides on PGs released from h-CM cells are shown. **B**: Those secreted by CHO-B_2_ cells are shown. These data are from a representative experiment for each cell type that were reproduced in several additional studies. The agonist potency values (EC_50_s, mean ± SEMs) obtained from three to six such experiments for h-CM cells and CHO-B_2_ cells are shown in Table 2. Agonist activity data for HOE140 and WIN-64338 are also listed in Table 2, and as expected, the latter antagonists on their own did not induce PGs release in either cell type.

Since the human TM cells exposed to various receptor agonists, including BK, produced various PGs [33], we decided to explore whether a similar phenomenon occurred in h-CM cells. The basal levels of total PGs released were lower in h-CM cells than in the CHO-B_2_ cells (mean of 56 versus 165 pg/ml of total PGs, respectively). BK and its analogs stimulated the generation and secretion of total PGs into the extracellular medium in a concentration-dependent manner in h-CM cells (Figure 5A; Table 2) and in CHO-B_2_ (Figure 5B; Table 2) cells, with potencies and rank order of activity similar to their [Ca^2+^]_i_-mobilization effects (Table 1). However, the peptides were generally less potent in the PG release assays than in the [Ca^2+^]_i_ mobilization assays. BK induced up to a sevenfold increase in the levels of total PGs relative to basal levels in h-CM cells depending on the donor cells, while a 4.9-fold elevation in extracellular PGs was observed in the CHO-B_2_ cells. BK-induced total PGs production in the h-CM cells was, for example, 393.3±24.2 pg/ml (n=8) at concentrations ranging from 0.1 to 10 µM. In CHO-B_2_ cells, the amounts secreted were 913.6±47.6 pg/ml (n=6). PGE_2_ levels were the most elevated followed by PGF_2α_ in response to BK, and no PGD_2_ was detected in h-CM cells with or without BK treatment. The potencies of BK and BK-related peptides at promoting PGE_2_ and PGF_2α_ release in h-CM cells were similar (Table 3) and compared well with their effects on total PG release (Table 2). CHO-B_2_ cells were much more responsive to the peptides than h-CM cells since the agonist potencies for initiating secretion of PGE_2_, PGF_2α_, and PGD_2_ were 100–1000 fold greater (Tables 3).

**Table 2:** Agonist potencies of BK and related peptides and other compounds in production and release of total PGs in primary h-CM cells and in CHO-B2 cells

**Compound**	**Total PGs Production in** **h-CM cells** **(EC_50_, nM)**	**Total PGs production in CHO-B2 cells** **(EC_50_, pM)**
BK	8.6±2.4	26.3±6.6
Hyp^3^-BK	10.9±6.1	68.3±36.7
Lys-BK	13.1±4.9	66.3±52.2
Met-Lys-BK	23.3±9.5	104.0±11.4
RMP-7	24.5±5.5	31.9±9.2
Des-Arg^9^-BK	>1,000	>100,000
Sar-[D-Phe^9^ ]-Des-Arg^9^-BK	>1,000	>1,000
HOE-140	>10,000	>10,000
(R)-WIN-64338	>10,000	>10,000
(S)-WIN-64338	>10,000	>10,000

Data are mean±SEM from 3 to 6 independent experiments for each cell type and for each compound tested. For reference, the amount of total PGs secreted by h-CM cells in response to 100 nM to 10 µM BK was 393.3±24.2 pg/ml, while in CHO-B2 cells the amounts were 913.6±47.6 pg/ml.

**Table 3:** PGE_2_, PGF_2α_ and PGD_2_ release induced by various agents in h-CM and CHO-B2 cells

**Compound**	**PGE_2_ production in h-CM cells** **(EC_50_, nM)**	**PGF_2α_ production in** **h-CM cells** **(EC_50_, nM)**
BK	8.9±0.2	15.9±9.2
Hyp^3^-BK	10.1±4.2	9.2±5.3
Lys-BK	10.3±5.9	35.6±16.1
Met-Lys-BK	32.2±9.0	35.6±16.1
RMP-7	27.2±9.8	18.4±5.2
Des-Arg^9^-BK	>1,000	>1,000
HOE-140	>10,000	>10,000
(R)-WIN-64338	>10,000	>10,000
(S)-WIN-64338	>10,000	>10,000

**Compound**	**PGE_2_ production in CHO-B2 cells** **(EC_50_, pM)**	**PGF_2α_ production in CHO-B2 cells** **(EC_50_, pM)**	**PGD_2_ production in CHO-B2 cells** **(EC_50_, pM)**
BK	32.8±9.03	32.1±12.2	11.3; 181
RMP-7	29.4±16.2	26.5±12.8	nd
Hyp^3^-BK	30.7±18.5	59.3±37.3	30.4; 58.2
Lys-BK	67.5±27.5	71.2±25.1	26.3; 64.6
Met-Lys-BK	139.0±51.3	106.0±70.5	78.8; 96.6
Des-Arg^9^-BK	>100,000	>100,000	>100,000
HOE-140	>10,000	>10,000	nd
(R)-WIN-64338	>10,000	>10,000	nd
(S)-WIN-64338	>10,000	>10,000	nd

Data are mean ± SEM from 3 to 6 experiments for each cell type; nd represents not determined.

**Figure 6:**
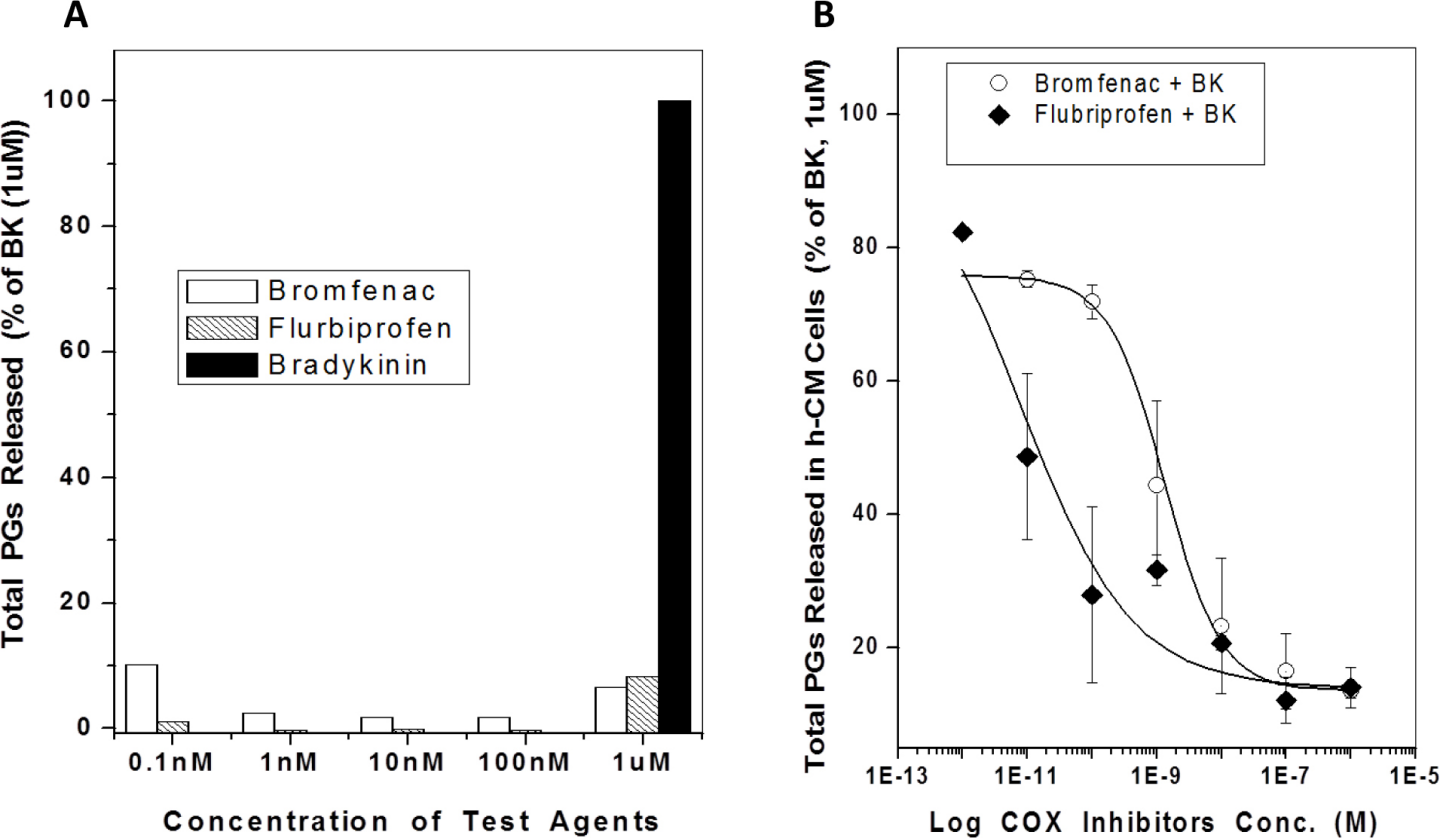
The effects of cyclooxygenase (COX) inhibitors on total PG release from human ciliary muscle (h-CM) cells. Concentration-dependent effects of flurbiprofen and bromfenac on their own on PG secretion was studied along with the positive control agent BK. Both COX inhibitors inhibited the background level of total PGs released from h-CM cells in an apparent concentration-dependent manner up to 100 nM, whereas BK (1 µM) substantially increased the PGs secreted above the basal levels (Figure 6A; data from a single experiment). Pretreatment of h-CM cells with various concentrations of the COX inhibitors before challenge with 1 µM BK resulted in a concentration-dependent reduction in total PGs synthesized and secreted, with flurbiprofen much more potent than bromfenac (Figure 6B). Data are mean ± SEM from three experiments for each inhibitor (Figure 6B).

**Figure 7:**
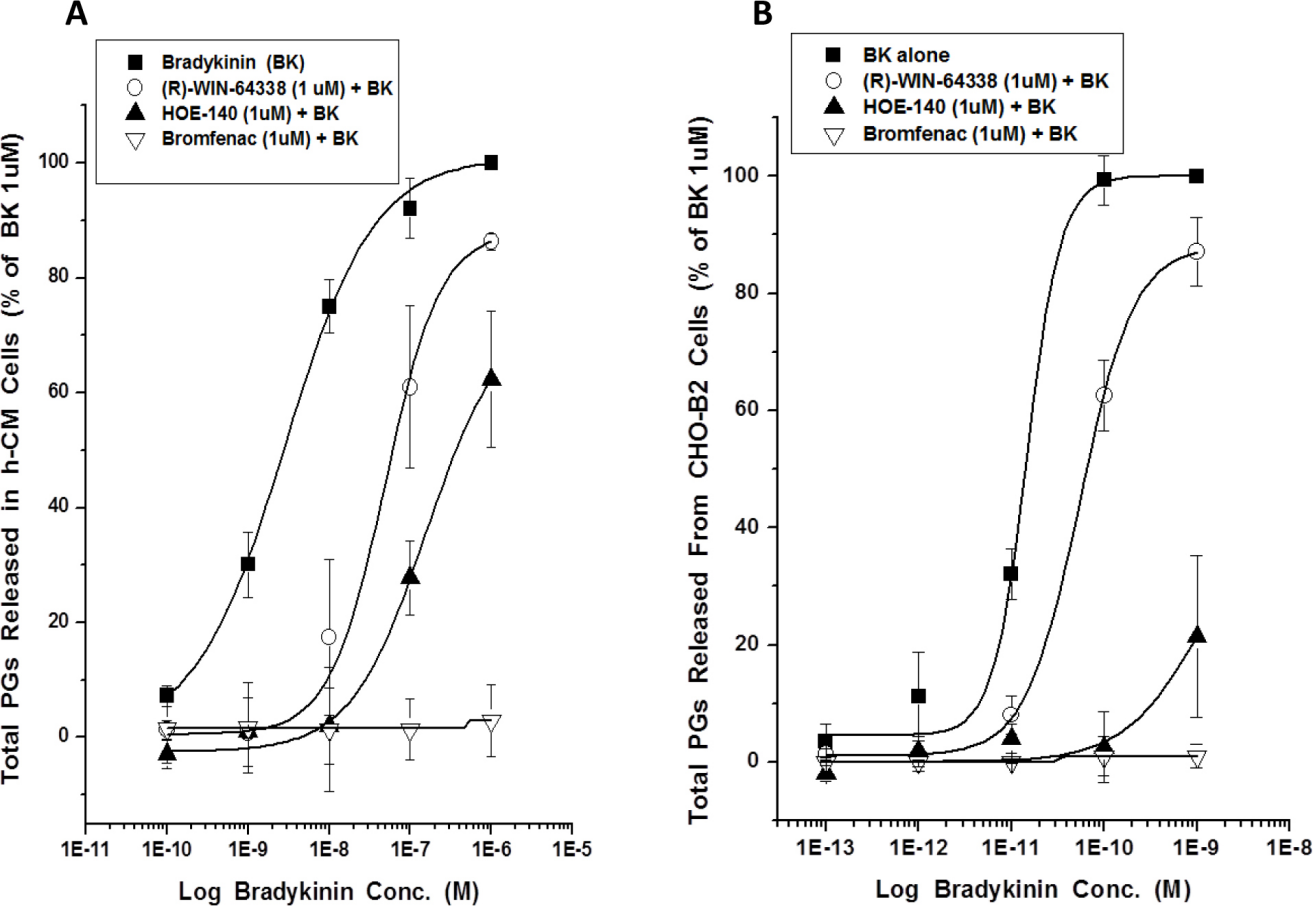
Effect of two B_2_-receptor antagonists and a cyclooxygenase (COX) inhibitor on release of total PGs from h-CM cells and CHO-B_2_ cells. **A**: Concentration-response studies with BK were conducted in primary h-CM cells in the absence and presence of the fixed concentration of receptor antagonists and enzyme inhibitor (all at 1 µM final) preincubated with the cells for 5 min before BK was added. Note that, although bromfenac completely abolished PG synthesis and release, WIN-64338 and HOE-140 caused a right-ward shift of the BK-induced PG release, indicative of competitive inhibition at the B_2_ receptor, with HOE-140 more potent than WIN-64338. **B**: The results obtained using CHO-B_2_ cells and the same experimental protocol as described above for the h-CM cells. Both sets of data are mean±SEMs from three experiments for each cell type.

Even though various B_2_ antagonists (HOE-140; (S)-WIN-64338; (R)-WIN-64338) and inhibitors of cyclooxygenases (COX; flurbiprofen; bromfenac) on their own failed to influence PG production in h-CM and CHO-B_2_ cells (e.g., Figure 6A; Table 2), the antagonists and inhibitors blocked the BK-induced total PGs production in both cell types (Figure 6B, Figure 7A,B).

### Measurement of nitric oxide

In limited experiments, we showed that while the NO donor sodium nitroprusside (1 mM) increased NO in h-CM cells by ninefold above basal levels, BK (0.1 and 1 µM) failed to have any effect on NO levels (data not shown).

### Extracellular signal-regulated kinase-1/2 phosphorylation and pro-matrix metalloproteinase production

Initial assay development included a cell number and concentration-response titration of BK stimulation in the h-CM and CHO-B_2_ cells with subsequent evaluation of phospho-ERK1/2 using the HTRF kit. The basal background levels of ERK1/2 phosphorylation were much higher in the CHO-B_2_ cells, and we did not obtain a meaningful response to BK in these cells. However, the h-CM cells yielded a better signal-to-noise response ratio and lower basal background levels of phosphorylation. The stimulation of ERK1/2 phosphorylation was 1.9±0.3 fold (n=3) in the presence of 100 nM BK (or RMP-7) compared to the basal ERK1/2 phosphorylation level using 50k h-CM cells/well and a 10 min incubation period. In subsequent concentration-response experiments, we found the potency of BK ranged between 20 nM and 80 nM, with a maximal effect at 1 µM, and HOE-140 (100 nM) reduced the ERK1/2 phosphorylation induced by BK down to basal levels.

Since BK and related peptides released PGs from h-CM cells (see above), we expected that the generation of MMPs would follow as has been previously demonstrated for FP-receptor agonists PGF_2α_ and PHXA85 in h-CM cells [29,33]. Accordingly, BK (10 nM to 1 µM for 24 h at 37 ^○^C) stimulated release of pro-MMP-1 (3.12±0.46-fold above basal level, n=6), pro-MMP-2 (1.67±0.25-fold above basal level, n=6), and pro-MMP-3 (1.41±0.09-fold above basal level, n=5). Likewise, RMP-7 (1–10 µM) also increased the secretion of pro-MMP-1 and pro-MMP-2 1.4–2.1-fold above basal levels in h-CM cells following a 24 h incubation. In contrast, the peptide B_2_-receptor antagonist, HOE-140 (10 nM to 1 µM) alone, failed to alter levels of pro-MMP-1 (0.94±0.13-fold of basal level), pro-MMP-2 (0.87±0.1-fold of basal level), and pro-MMP-3 (0.95±0.1-fold of basal level; all n=6) in the h-CM cells.

### Intraocular pressure changes induced by kinins

**Figure 8:**
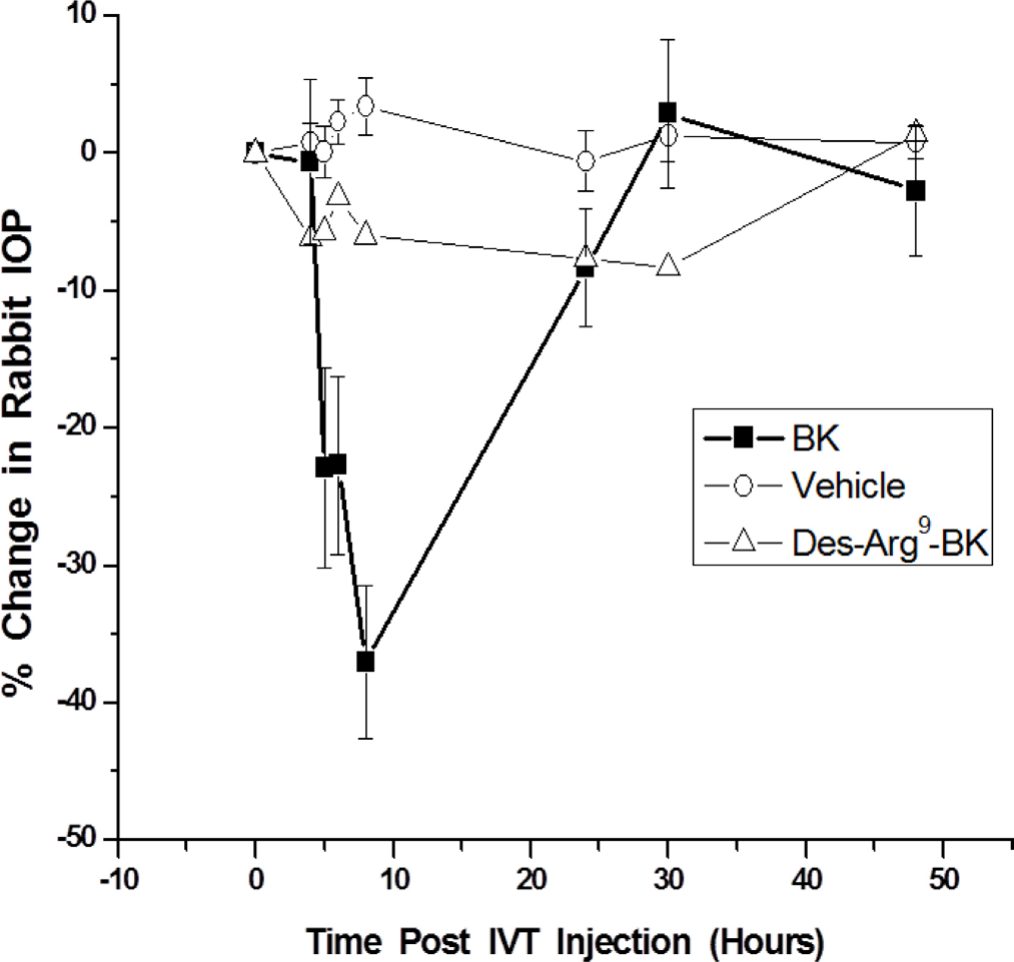
Time-course of changes in rabbit intraocular pressure (IOP) with different kinin peptide agonists. Three groups of Dutch-Belt rabbits (n=7–10/group) were injected ivt in one eye with either vehicle or 50 µg of BK or 50 µg of Des-Arg^9^-BK dissolved in the vehicle and the IOP monitored over time. Data are mean±SEM.

We studied the effects of topical ocularly administered BK (100 µg) in the ocular normotensive and hypertensive eyes of conscious cynomolgus monkeys to link the B_2_-receptor immunoreactivity observations in the CM and non-pigmented ciliary epithelium (NPCE) tissues in this species. However, due to the poor ocular penetration of BK across the monkey cornea and conjunctiva, and the metabolic instability of this peptide, no alteration in IOP was observed in the monkey eyes (Table 4). In contrast, when BK (50 µg) was injected into the vitreous of the Dutch-Belt rabbits, a time-dependent reduction in IOP was observed, with a maximal 37.0±5.6% reduction 8 h post-injection (Figure 8). However, neither the vehicle nor Des-Arg^9^-BK (50 µg) injected ivt influenced rabbit IOP (Figure 8). Thus, the IOP-lowering effect of BK in the rabbits was specific and mediated by the B_2_ receptors.

**Table 4:** Effects of topical ocular bradykinin on IOP of Cynomolgus monkey eyes

Cynomolgus monkey studies	**Monkey IOP changes after topical ocular dosing**		
	Time post- administration (hours)	IOP (mmHg)	IOP change (% of control)
***Normal pressure monkey eyes***	0 h	27.1±1.0	0
BK (100 µg) group	1 h	27.1±1.1	0.2±2.5%
BK (100 µg) group	3 h	26.6±0.7	−1.0±2.9%
BK (100 µg) group	6 h	27.2±1.1	0.8±2.3%
BK (100 µg) group	24 h	27.8±1.4	2.4±3.3%
***Ocular hypertensive monkey eyes***	0 h	36.8±1.6	0
BK (100 µg) group	1 h	35.9±1.8	−2.6±1.5%
BK (100 µg) group	3 h	35.7±1.6	−2.7±3.3%
BK (100 µg) group	6 h	36.6±1.7	−0.4±3.0%
BK (100 µg) group	24 h	35.3±1.6	−4.1±2.3%
*Cabergoline* (50 µg) group*	*0 h*	*34.7±3.0*	*0*
	*1 h*	*nd*	*-16.0%*
	*3 h*	*nd*	*-30.4%*
	*7 h*	*nd*	*-28.8%*
	*24 h*	*nd*	*-27.3%*

Data are mean ± SEM from twelve conscious Cynomolgus monkeys with one normotensive (control eye) and one eye rendered hypertensive with laser trabeculoplasty. * Data for cabergoline (positive control agent) are from Ref. Thirty for comparison with another topical ocularly administered compound in the ocular hypertensive eyes of nine conscious Cynomolgus monkeys; nd represents not determined.

## Discussion

A multidisciplinary approach was used to demonstrate the presence of various elements of the kallikrein/kinin system in humans, and where possible in Cynomolgus monkeys, ocular tissues/cells, and to explore the pharmacological aspects of the signal transduction systems in h-CM cells (and in CHO-B_2_ cells) using various biochemical techniques. Regarding the human eye, previous studies using reverse transcriptase (RT)–PCR and in situ hybridization demonstrated the presence of mRNAs for the B_2_ receptor in the ciliary body, but without showing sub-regional tissue/cellular localization [19,22]. We extended these observations by documenting a high level of B_2_-receptor immunoreactivity associated with human (Figure 2A) and Cynomolgus monkey (Figure 2C) CM and non-pigmented ciliary epithelial cells. In addition, we extensively characterized the biochemical pharmacology of the BK receptor-effector coupling in isolated h-CM cells. Thus, we demonstrated that BK, Hyp^3^-BK, Lys-BK, and RMP-7 potently and efficaciously stimulated the mobilization of [Ca^2+^]_i_ in h-CM cells with single-digit nanomolar potency values, while Met-Lys-BK and Des-Arg^9^-BK were markedly less potent (Figure 3A,B; Table 1). This pharmacological profile of the functional agonist responses in h-CM cells matched well the pharmacology of the agonist-induced production of IPs in human trabecular meshwork (h-TM) cells [6], and that of the ability of these peptides to compete for the agonist binding sites on human cloned B_2_ receptors labeled with [^3^H]-BK (Table 1). In addition to the agonist rank order of potencies, since the BK-induced [Ca^2+^]_i_ mobilization in h-CM cells (and that in h-TM cells in preliminary experiments; Table 1) was blocked by HOE-140 and WIN-64338 (Figure 3C,D; Table 1) [3,34,35], a B_2_-receptor pharmacological signature was ascribed to the functional h-CM cell BK receptor.

The possible sources of the elevated [Ca^2+^]_i_ in response to BK in h-CM cells were explored in some detail in subsequent experiments. Since removing extracellular Ca^2+^ with 1–2 mM EGTA totally abolished the BK-induced [Ca^2+^]_i_ mobilization (Figure 4A), at least some, if not all, of the Ca^2+^ detected inside the cells was apparently originating from the extracellular space. However, EGTA is known to chelate some of the intracellular Ca^2+^ from organelles such as the ER and mitochondria [7,36], and thus determining the involvement of the intracellular compartments in the BK-induced responses was considered important. Thus, when PLC was inhibited by preincubation with U73122 (Figure 4B), the effects of BK were diminished in a concentration-dependent manner indicating that indeed generating IPs by BK was also necessary to elevate the concentration of [Ca^2+^]_i_ in h-CM cells akin to that observed in many different cell types [4,20,21,33,37-39], including h-TM cells [6,15]. The fact that the cytosolic Ca^2+^ increase observed in response to BK in h-CM cells partially originated from the ER was demonstrated next by pretreating the cells with thapsigargin, an inhibitor of the Ca^2+^-ATPase that is responsible for pumping free Ca^2+^ into the ER from the cytoplasm [32] before exposure to BK. Thus, as expected thapsigargin partially depleted the ER store of Ca^2+^, and the response to BK in h-CM cells was blunted but not abolished (Figure 4C), indicating that extracellular Ca^2+^ was mobilized by BK when the supply was limited from the ER. Interestingly, when extracellular Ca^2+^ was chelated by 1 mM EGTA and the ER pool of free Ca^2+^ depleted, BK was still enhanced the [Ca^2+^]_i_, perhaps by activating release from other intracellular organelles of h-CM cells such as the Golgi body and/or mitochondria as has been suggested for other cell types and tissues such as cardiac, skeletal, and smooth muscles [32].

The cell membrane serves as a major barrier between the intra- and extracellular compartments, and indeed Ca^2+^ homeostasis is a key function of many types of channels located in the membranes. Although some of these channels are activated or modulated directly by extracellular signaling compounds (ligand-gated), others are modulated by second messengers generated by ligand-receptor activation [7]. In additional studies (data not shown), we demonstrated that RMP-7 transiently opened voltage-gated Ca^2+^ channels in human embryonic kidney cells transfected with human wild-type sub-units of L-type voltage gated calcium channels (VGCCs; α1C [Cav1.2], β1b, and α2δ subunits) and that express native B_2_ receptors, to permit entry of extracellular Ca^2+^ to the cell interior. These collective data indicate that BK and its close analogs can mobilize Ca^2+^ from the intracellular organelles (ER, Golgi, mitochondria) as well as from the extracellular space to raise the observed concentration of [Ca^2+^]_i_ in response to B_2_-receptor stimulation (Figures 3,4), observations akin to those reported for bovine TM cells [40].

The B_2_ receptor has been shown to couple to a multitude of cell signaling pathways in various cells [3]. Central to these mechanisms appears to be the increased [Ca^2+^]_i_ that BK produces via the IP_3_-sensitive and ligand-gated Ca^2+^-channel activation [3,7]. Although BK can elicit production of NO and cGMP in certain endothelial and neuronal cells [3,28], BK failed to do so in h-CM cells. However, as shown in the h-TM cells [33], human corneal epithelial cells [21], and other cell types [3,41,42], BK and its close analogs (except the B_1_-receptor agonist Des-Arg^9^-BK) were relatively potent inducers of PG synthesis and secretion from h-CM and CHO-B_2_ cells, (Figures 5A, 5B; Table 2, Table 3). Although the absolute potency values of the BK-related agonists in the PG release assays differed slightly from their potencies for increasing levels of [Ca^2+^]_i_ in h-CM cells, an observation also noted for CHO-B_2_ cells, the overall rank order of activity of the peptides was similar (Tables 1, Table 2). The lower potency in the PG secretion assays could be partially due to the degradation of the peptides during the long incubation with the cells whereas the Ca^2+^-response assay requires a few minutes’ exposure to the cells. Regardless, however, the agonist potencies we obtained in the PG release assays in both cell types indicated a B_2_-receptor pharmacology akin to the [Ca^2+^]_i_ mobilization read-out. This was further confirmed by the potent antagonism of the BK-induced PG synthesis and secretion from h-CM cells (Figure 7A) and CHO-B_2_ cells (Figure 7B) by HOE-140 and WIN-64338, two well-known B_2_-selective antagonists [3,6,20,35]. The involvement of COX enzymes in producing PGs in h-CM and CHO-B_2_ cells in response to BK was demonstrated by the near-complete abolition of this response by two COX inhibitors, flurbiprofen and bromfenac (Figure 6A, B; Figure 7A,B), with flurbiprofen the more potent inhibitor (Figure 6B). Since the latter inhibitor equally blocks COX-1 and COX-2, whereas bromfenac is COX-2-selective [43], our data suggest the participation of both enzymes in the BK-induced PG production in h-CM cells.

The downstream effects of PG formation and secretion from h-CM cells in response to BK was of interest since at least PGF_2α_ can activate MMP production via an ERK-dependent mechanism [29,44]. Indeed, since FP receptors are expressed by h-CM cells [45], it was interesting to find that BK and RMP-7 relatively potently increased ERK1/2 phosphorylation in h-CM cells, and perhaps this was then responsible for the production of pro-MMPs-1, -2, and -3 detected in response to BK and RMP-7 (see Results).

Despite the presence of the kallikrein/kinin enzyme and receptor system in the h-CM tissue and cells, with B_2_-receptor immunoreactivity also found in the cynomolgus monkey CM (Figure 2B), the physiologic roles of BK and its receptors in this tissue and cells remain to be elucidated. Indeed, there are conflicting reports in the literature regarding the functions and involvement of BK in modulating IOP in various animal models. Thus, while topical dosing of BK in albino rabbits [8] and intravenous infusion of BK apparently lowered IOP [9], intracameral injection of BK raised IOP, caused intense miosis [10,14], and increased aqueous humor inflow and outflow [11]. Furthermore, BK either had no effect on aqueous humor outflow (no ciliary muscle retrodisplacement) or decreased outflow (with ciliary muscle retrodisplacement) in cynomolgus monkey eyes upon intracameral injection of BK into the eye [12]. Additionally, in isolated perfused human and bovine anterior eye segments, BK decreased outflow facility [15], while another group has recently demonstrated an apparent increase in outflow in isolated perfused bovine eyes [16]. In our animal studies, we also encountered differing results such that topically applied BK failed to modulate IOP in conscious ocular normotensive and hypertensive cynomolgus monkey eyes (Table 4), whereas direct delivery of BK (50 µg) into the vitreous of the rabbit produced a robust lowering of the IOP 5–8 h post injection (Figure 8). The fact that 50 µg of the B_1_-receptor agonist Des-Arg^9^-BK failed to reduce rabbit IOP (Figure 8) indicated that indeed the BK-induced ocular hypotension following ivt injection of BK was B_2_ receptor mediated as all the biochemical signal transduction processes we studied in h-CM cells and as described above. Whether similar IOP reduction is caused by ivt-injected BK in monkey eyes remains to be determined in future studies, but the high homology of the B_2_ receptor among several mammalian species [46] suggests that this is quite likely. Regardless, however, the presence of the kallikrein/kinin system, including functionally active B_2_ receptors in the CM of human and monkey eyes, strongly suggests that BK plays a role in fluid hydrodynamics to modulate IOP. Further work in this arena seems warranted to expand our knowledge on the roles of the endogenous peptides, BK and Lys-BK, in anterior chamber functions.

In conclusion, these studies have collectively demonstrated the presence of B_2_-receptor protein and BK binding sites on human CM and cells isolated from the latter tissue. Furthermore, these receptor proteins were functionally coupled to PLC to produce various intracellular second messengers, including [Ca^2+^]_i_, that appears to be originating mainly from the ER. Although NO appeared not to be involved, the activation of cyclooxygenases and ERK1/2 by BK and related peptides in h-CM cells was evident. The pharmacological attributes of the Ca^2+^-mobilizing and PG secretion activities induced by BK and related peptides (e.g. RMP-7) [47], and their blockade by various antagonists, clearly confirmed the receptor mediating these responses to be the B_2_ subtype. We can therefore use the results of our mechanistic studies to build a potential sequence of events as follows: thus, activation of the B_2_ receptor in h-CM cells activates PLC, which generates various inositol phosphates that then cause the release of Ca^2+^ from the ER. This elevated Ca^2+^ then stimulates ERK1/2 and COX enzymes to liberate various PGs (mostly PGE_2_ but also PGF_2α_) into the extracellular space, and these PGs stimulate various PG-receptor sub-types to liberate MMPs [29,44,45]. The latter subsequently degrade the extracellular matrix between CM bundles and sclera (perhaps also in the TM) to enhance the uveoscleral outflow (and conventional outflow) of aqueous humor that ultimately results in reduced IOP.
